# The role of social attraction and its link with boldness in the collective movements of three-spined sticklebacks

**DOI:** 10.1016/j.anbehav.2014.11.004

**Published:** 2015-01

**Authors:** Jolle W. Jolles, Adeline Fleetwood-Wilson, Shinnosuke Nakayama, Martin C. Stumpe, Rufus A. Johnstone, Andrea Manica

**Affiliations:** a Department of Zoology, University of Cambridge, Cambridge, U.K.; b Department of Biology and Ecology of Fishes, Leibniz Institute of Freshwater Ecology and Inland Fisheries, Berlin, Germany; c Albrecht Daniel Thaer Institute of Agriculture and Horticulture, Humboldt-Universität zu Berlin, Berlin, Germany; d AnTracks Computer Vision Systems, Mountain View, CA, U.S.A.

**Keywords:** animal personality, behavioural syndrome, boldness, collective behaviour, coordination, leadership, sociability, social attraction, three-spined stickleback

## Abstract

Social animals must time and coordinate their behaviour to ensure the benefits of grouping, resulting in collective movements and the potential emergence of leaders and followers. However, individuals often differ consistently from one another in how they cope with their environment, a phenomenon known as animal personality, which may affect how individuals use coordination rules and requiring them to compromise. Here we tracked the movements of pairs of three-spined sticklebacks, *Gasterosteus aculeatus*, separated by a transparent partition that allowed them to observe and interact with one another in a context containing cover. Individuals differed consistently in their tendency to approach their partner's compartment during collective movements. The strength of this social attraction was positively correlated with the behavioural coordination between members of a pair but was negatively correlated with an individual's tendency to lead. Social attraction may form part of a broader behavioural syndrome as it was predicted by the boldness of an individual, measured in isolation prior to the observation of pairs, and by the boldness of the partner. We found that bolder fish, and those paired with bolder partners, tended to approach their partner's compartment less closely. These findings provide important insights into the mechanisms that govern the dynamics and functioning of social groups and the emergence and maintenance of consistent behavioural differences.

## Highlights

• We investigated social attraction during the collective behaviour of pairs of fish.
• Social attraction was positively correlated with better coordination in the pair.
• Individual leadership was negatively linked with social attraction.
• Greater boldness predicted lower social attraction, suggesting a behavioural syndrome.
• The boldness of the partner also influenced the tendency for social attraction.

Social animals may benefit from grouping due to reduced predation risk, earlier predator detection and greater foraging success (Krause & Ruxton, 2002; Pitcher & Parrish, 1993). At the same time, grouping may entail costs in the form of increased competition and predator attraction (Krause & Ruxton, 2002). To ensure that individuals reap the full benefits of grouping, they must coordinate their behaviour with other group members (Conradt & Roper, 2009; Van Vugt, 2006), resulting in collective movements and decisions (Couzin et al., 2011; Miller, Garnier, Hartnett, & Couzin, 2013) and the possible emergence of leaders and followers (King, Johnson, & Van Vugt, 2009; Krause & Ruxton, 2002). Focus on the mechanisms that govern such collective behaviour may increase our understanding of the social organization and dynamics within and across groups, from aggregating insects to human societies (Conradt & List, 2009; King et al., 2009).

Group movements and decisions can often be explained by individuals following simple rules (Couzin & Krause, 2003; Couzin, Krause, Franks, & Levin, 2005; Sumpter, 2006). However, individuals often behave consistently different from one another, now mostly referred to as animal personality (Réale, Dingemanse, Kazem, & Wright, 2010; Réale, Reader, Sol, McDougall, & Dingemanse, 2007; Sih, Bell, & Johnson, 2004), with large potential consequences for the functioning and structure of social groups (Wolf & Krause, 2014). Particularly relevant in the context of collective behaviour is boldness, i.e. individual variation in the tendency to take risks. Bolder individuals may be more likely to lead, so as to maximize their foraging opportunities (Biro & Stamps, 2008; Jolles, Ostojić, & Clayton, 2013; Kurvers, Prins, et al., 2010), whereas shy individuals may be more likely to group (Bell & Sih, 2007) and respond to conspecifics (Croft et al., 2009; Harcourt, Biau, Johnstone, & Manica, 2010; Kurvers, van Oers, et al., 2010; Pike, Samanta, Lindström, & Royle, 2008; Trompf & Brown, 2014) for antipredator benefits (Krause & Ruxton, 2002). Although previous work has confirmed that bolder individuals are more likely to take the lead and shy individuals are more likely to follow (Harcourt, Ang, Sweetman, Johnstone, & Manica, 2009; Jolles et al., 2014; Kurvers et al., 2009; Nakayama, Harcourt, Johnstone, & Manica, 2012), the impact of boldness on social attraction during collective movement remains unclear.

Previous work that focused on the tendency for individuals to approach and interact with conspecifics, i.e. sociability, has revealed large ecological and evolutionary implications (Cote & Clobert, 2007; Cote, Fogarty, Weinersmith, Brodin, & Sih, 2010; Réale et al., 2007). Also in the context of collective behaviour (Cote, Fogarty, & Sih, 2012; Dingemanse & Réale, 2005; Réale et al., 2007), sociable individuals have more and stronger social associations (Croft et al., 2009), have stronger grouping preferences (Cote et al., 2012), and play a key role in group exploration (Brown & Irving, 2014). However, studies typically measure the tendency of individuals to approach a static group of conspecifics or a larger over a smaller one (see Wright & Krause, 2006). Not only is this less relevant to the natural situation, where individuals can interact and respond to one another (see also Miller & Gerlai, 2007), but also the mechanistic role of sociability in coordination and leadership and the potential effects of social feedback remain unclear (Miller & Gerlai, 2008; Wilson, Krause, Dingemanse, & Krause, 2013). For example, sociability may be linked to the distance regulation between individuals (Krause, Hoare, Krause, Hemelrijk, & Rubenstein, 2000; Pitcher & Parrish, 1993), and thereby affect leading and following behaviour. Our aim in this study was to explore social attraction, i.e. the tendency for individuals to approach a partner, in the context of joint movement of pairs of stickleback fish. This allowed us to assess the impact of social attraction on collective behaviour and look at the role played by social feedback in a similar way to recent studies on boldness and leadership (Jolles et al., 2014; Nakayama, Stumpe, Manica, & Johnstone, 2013; Pettit, Perna, Biro, & Sumpter, 2013; Ward et al., 2013).

We repeatedly observed three-spined sticklebacks, *Gasterosteus aculeatus*, in a context in which they could rest under cover or explore an open, potentially risky environment. We first assessed the boldness of all fish by recording their behaviour in isolation, after which we subjected them to the same environment again but this time allowing observation of a conspecific through a transparent partition. By tracking the movements of both fish in and out of cover and determining their tendency to approach their partner's compartment, we aimed to determine (1) whether individuals are consistent in the strength of social attraction they exhibit, in an ecologically relevant setting, (2) what link may exist between the tendency for social attraction and boldness, (3) whether social attraction is influenced by the personality of a partner as well as that of the focal individual and (4) how social attraction varies during collective movements (when both fish are out of cover) compared to when fish are out alone, either during the initiation and return of such movements or during solitary trips. This approach of subjecting fish to a dynamic social context in which they can move with conspecifics as well as rest under cover provides a unique opportunity to describe important new aspects of sociability and risk-taking behaviour and their role in the mechanisms underlying collective behaviour.

## Methods

### Overview

**Figure 1:**
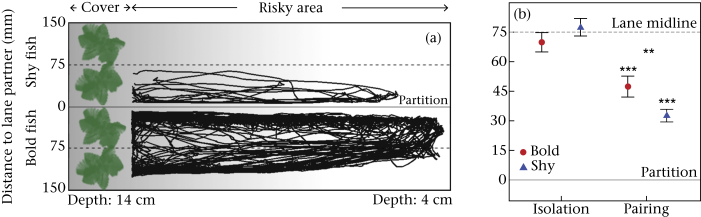
(a) Schematic overview of the experimental tank with a transparent partition, with all movement trajectories (black lines) of a representative pair of fish when not in the cover area (15 cm length) during the first day of the pairing stage. (b) Distance to the partner's lane (mm) for focal fish bolder and shyer than their partner, during the isolation (*N* = 19; *N* = 19) and pairing stages (*N* = 14; *N* = 15). ** *P* < 0.01; *** *P* < 0.005.

Ninety-six fish were tested repeatedly in a task in which they could either rest under cover or explore an open, potentially risky environment (risk-taking task). Two such task compartments were positioned adjacent to one another and separated by either an opaque or a transparent partition (see Fig. 1). First, we tested fish individually in the compartments separated by an opaque partition to investigate their propensity to explore a risky area when alone (‘isolation stage’). Second, we tested fish in the risk-taking task again but now with the compartments separated by a transparent partition, thus allowing fish to see each other and interact (‘pairing stage’). By testing fish twice in each stage we were able to get individual consistency scores of risk-taking behaviour when alone (boldness) and their tendency to approach the partner's compartment when together (social attraction). Third, we tested an additional 16 fish using the same procedure as above, but with a transparent instead of an opaque partition and an empty adjacent compartment during the isolation stage. This allowed us to ensure differences in behaviour between the two stages were not simply due to the transparency of the partition.

### Subjects and Housing

We collected three-spined sticklebacks during the summers of 2010–2012 using a sweep net from a small branch of the River Cam (Cambridge, U.K.). Fish were taken from a single population to minimize population-specific genetic effects that may influence personality (Bell, 2005). After collection, fish were immediately housed in a temperature-controlled laboratory (14 ± 1 °C) with constant light regime (lights on from 0900 to 1900 hours), and kept in large, glass social-housing tanks (120 × 60 cm and 60 cm high) with artificial plants, aeration and under-gravel filtration. During this period before experiments, fish were fed frozen bloodworms (chironomid larvae) ad libitum once daily. As the temperature and photoperiod regime in the laboratory prevented the fish from becoming sexually mature (Borg, Bornestaf, & Hellqvist, 2004), we did not sex the fish.

We performed our experiments with four batches of fish (*N* = 96 total), which were about 6 months old at the time of testing. After an acclimatization period of at least 1 month, for each batch we randomly selected fish from the social-housing tanks controlling for size (mean ± SE: 44 ± 1 mm from tip of snout to caudal peduncle). Fish were subsequently housed in custom holding tanks (60 × 30 cm and 40 cm high) lined with gravel and divided lengthwise into six compartments (30 × 12 cm; 15 cm deep). Five of the compartments contained an artificial plant; the remaining compartment contained an under-gravel filter and was not used to house fish. Fish were randomly allocated to compartments. To minimize potential stress effects that may be caused by isolation, the compartments were divided by perforated transparent Perspex partitions, thus allowing fish to receive chemical and visual cues from conspecifics. After the experiments, fish were kept in the laboratory and used for additional behavioural experiments. Animal care and experimental procedures were approved by the Animal Users Management Committee of the University of Cambridge under a nonregulated procedures regime because of the nonintrusive and observational nature of our work.

### Experimental Set-up

To investigate fish's willingness to take risks as well as their tendency to approach conspecifics during collective behaviour, we used a tank set-up as previously used in our laboratory for similar experiments (Harcourt et al., 2009; Jolles et al. 2014; Nakayama et al. 2012). In short, experiments were carried out in four identical experimental tanks (70 × 30 cm and 30 cm high), each divided by either an opaque or a transparent Perspex partition to create two long compartments (15 cm wide). Each compartment was lined with gravel in a slope ranging from a deep area (14 cm deep) that contained an artificial plant to an increasingly shallow ‘exposed’ area (4 cm deep at the other side, see Fig. 1a). Only when fish had fully emerged from the deep ‘covered’ area (15 cm from the back of the compartment) did we define them to be ‘out of cover’. This set-up reflects the ecologically relevant situation in which fish can either rest in a safe place or explore a risky area (in search of potential food). Fish prefer to spend time under cover but, even in the absence of food, keep making regular trips out of cover to explore the exposed area. Fish have different preferences for how frequently and for how long they leave cover, yet try to coordinate their behaviour and shoal together, generating a conflict on the timing of leaving and returning to cover. Although the partition prevented the transfer of chemical cues, fish could see and interact with one another when we used the transparent partition. The other three sides of the compartments were covered by white Perspex to minimize any outside disturbances. When we were not running experiments, the water of the experimental tanks was oxygenated using airstones. An HD camera (Camileo X100, Toshiba Corporation, Japan) positioned above each tank was used to record the movements of the fish.

### Experimental Procedure

The first 3 days of the experimental period were used to acclimate the fish to their individual housing tanks. Subsequently, on the next 2 days, fish were tested for 1 h per day in the compartments separated by an opaque partition (‘isolation stage’). This allowed us to get a boldness score for each fish: consistency in the proportion of time out of cover. After a rest day, fish were again tested in the compartments for 2 days, 1 h per day, but now with the compartments separated by a transparent partition, thus allowing fish to see each other and interact (‘pairing stage’). Fish in adjacent compartments were the same for both days of the pairing stage. This testing of fish in ‘pairs’ allowed us to get social attraction scores for each fish based on their tendency to approach their partner's compartment (see Data Analysis for details), and enabled us to investigate the link between boldness and social attraction and their role during solitary and collective behaviour.

For each session, fish were transferred to the deep end of the tank using a dip net and allowed to acclimate for 7 min before we filmed their movements. Fish were tested in a random order and randomly assigned to tanks and tank compartments. To avoid nonindependence in our analyses, we randomly selected one fish in each pair (*N* = 48) as the focal individual and the other as its partner. During the experimental period fish were fed one bloodworm at the end of each day to standardize hunger levels. No food was provided during the experimental sessions and fish were thus not rewarded for leaving cover.

To ensure that any differences in behaviour between the isolation and pairing stages were not simply a response to the transparent partition itself (but rather the result of interactions between the two fish), we ran an additional control condition with naïve fish (*N* = 16). This ‘transparency control’ followed the same procedure as the main experiment (see above), but now during the isolation stage randomly selected focal fish (*N* = 8) were tested in a compartment separated by a transparent partition and with no fish in the adjacent compartment.

### Video Tracking

From the videos, we determined the exact position of all fish 10 times/s using automated motion-tracking software (AnTracks version 0.99, www.antracks.org), providing us with X and Y coordinates of each fish over time on a mm scale. For tracking, we used a background subtraction acquisition method that determines which pixels differ between a video and a background image created from a random 5 min period in each recording. For processing, we used Gauss subtraction, Gauss blur, dilate and final thresholding, with the parameters adjusted according to the light levels in each video to ensure the movements of the fish were tracked correctly. After tracking was complete, we visually checked all trajectories for each video, manually correcting any errors and joining discontinuous trips where the software had lost track of a fish for a few frames.

### Data Analysis

Data were analysed in R 3.0.2 (The R Foundation for Statistical Computing, Vienna, Austria, http://www.r-project.org). The positional coordinates of each fish were used to calculate the timing and proportion of time fish were out of cover and, as a proxy for social attraction, the fish's average distance to the partner's compartment (see Fig. 1a). To avoid nonindependence, the majority of analyses focused on the behaviour of the randomly predetermined focal fish, with certain analyses incorporating the personality of the partner (see below).

First, we quantified boldness, social attraction and coordination. Boldness scores were calculated as the average proportion of time individuals spent out of cover on both days of the isolation stage; and we confirmed that this was consistent using a Spearman rank correlation test. Previous work with the same experimental set-up has established that this behaviour is consistent over longer periods of time (Harcourt et al. 2009; Jolles & Manica, n.d.). A social attraction score was calculated as the average distance from the adjacent compartment during collective movements. To determine whether fish swam on average in the middle of their compartment, we used one-sample *t* tests and one-sample Wilcoxon signed-ranks tests. To get a measure of coordination within the pair, we used the same logic as used for estimating genetic linkage (Harcourt et al. 2009; Rands, Cowlishaw, Pettifor, Rowcliffe, & Johnstone, 2008), with scores reflecting the proportion of time both fish in a pair carried out the same behaviour relative to the time they carried out opposite behaviours.

Second, to determine whether fish changed their behaviour between the isolation and pairing stages, we used Wilcoxon signed-ranks tests and paired *t* tests, and we used a Spearman rank correlation test to investigate whether the change in risk-taking behaviour between the stages was linked to initial risk-taking behaviour. To compare social attraction of focal fish from the main experiment with fish from the transparency control group, we used an Independent Samples *t* test. Furthermore, as previous work has shown that the relative boldness of fish is important in social interactions (Harcourt et al. 2009; Nakayama et al. 2013), we compared the social attraction of focal fish that were bolder (‘bold focals’) and focal fish that were shyer than their partner (‘shy focals’). The relative boldness of focal fish to their partner ranged from −0.60 to +0.56 (shy focal fish: *N* = 15; bold focal fish: *N* = 14; mean ± SE = −0.03 ± 0.03). We excluded cases in which neither the focal fish nor its partner went out of cover during the isolation stage or the focal fish did not go out of cover during the pairing stage as no social attraction score could be calculated.

Third, to determine the role of boldness in risk-taking behaviour and social attraction during the pairing stage, we ran linear models with, respectively, the proportion of time spent out of cover (risk taking) and mean distance from the central partition (social attraction) by the focal individuals as response variables. As predictors, we fitted the boldness score of the focal individual, the boldness score of its partner and the interaction between them. To investigate how the overall social attraction of both fish in a pair affected their coordination, we ran a Spearman rank correlation; to determine its role in leadership behaviour we ran a linear model with, as response variable, the number of trips on which the focal individual led its partner, and the social attraction score of the focal fish and its partner as predictors. Leading was defined as the focal fish leaving cover and subsequently being joined by its partner. Minimal adequate models were obtained by backward stepwise elimination (i.e. sequentially dropping the least significant terms from the full model, until all terms in the model were significant). Statistics for nonsignificant terms were obtained by adding each nonsignificant term to the minimal model. Boldness and social attraction scores were square-root transformed to ensure homogeneity of variance, normality of errors and linearity.

Fourth, to further understand the dynamics behind social attraction, we investigated an individual's tendency to approach its partner's compartment both during ‘collective movements’, i.e. when both fish were out of cover and when fish were out alone. Fish could be out of cover alone during (1) the ‘initiation’ of collective movement: the focal fish left cover but was not joined by its partner; (2) the ‘return’ of collective movements: when the focal fish was still out after collective movement but the partner was already back in cover; and (3) ‘solo trips’: when the focal fish was out of cover and returned to cover without the partner coming out. We used paired *t* tests and Wilcoxon signed-ranks tests to compare social attraction across these different stages of collective movement. As body size did not have a significant effect on any of the measured behaviours and was not the focus of the present study, we have excluded it from our results. All results with *P* < 0.10 are reported as trends and *P* < 0.05 as significant. Means are quoted ± SE throughout.

## Results

### Risk-taking Behaviour

Fish were highly consistent in the proportion of time spent out of cover during the isolation stage (*r*_s_ = 0.65, *N* = 96, *P* < 0.001), providing the boldness score for each fish. Focal fish spent on average a similar amount of time out of cover during the isolation stage (0.10 ± 0.02) and the pairing stage (0.12 ± 0.02; *V* = 388, *P* = 0.145). However, there was a negative relationship between the initial time focal fish spent out of cover (i.e. their boldness) and their change in risk-taking behaviour across the stages (*r*_s_ = −0.37, *N* = 48, *P* = 0.010): bolder fish spent less time and shy fish more time out of cover during the pairing stage. The proportion of time focal fish spent out of cover during the pairing stage was predicted by a negative interaction between their own boldness and that of their partner (*F*_3,44_ = 9.22, *P* = 0.008, *R*^2^ = 0.39): although bolder fish in general spent more time out of cover than shyer fish, being paired with a relatively bold partner decreased this difference between bold and shy fish.

### Social Attraction: Stage Comparisons

**Figure 2:**
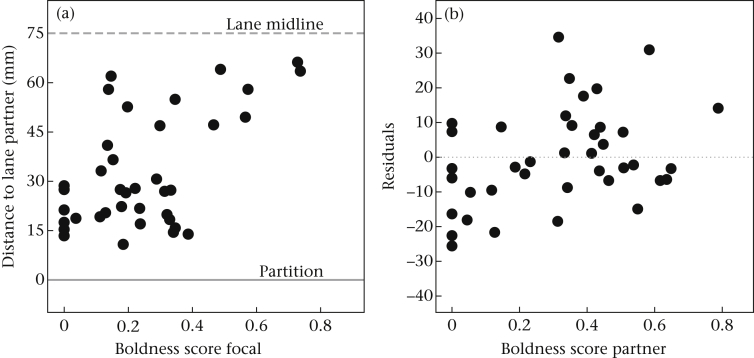
Distance of focal fish to their partner's lane (mm) in relation to (a) their own boldness and (b) the boldness of their partner during collective movements during the pairing stage. Boldness scores are square-root transformed.

**Figure 3:**
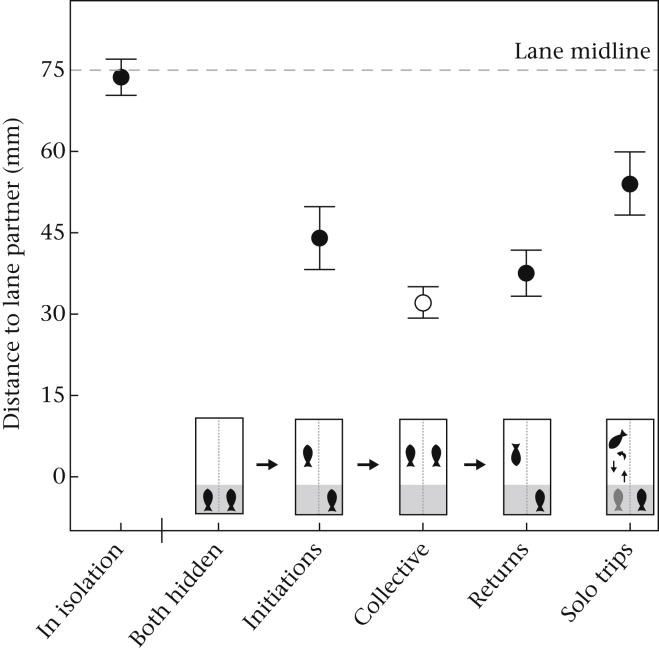
Plot showing the distance of focal fish to the lane of their partner (mm) during the isolation stage when out of cover, and during the pairing stage, separate for each of four possible states in which focal fish could be out of cover: when focal fish initiated trips later joined by their partner (initiations), when focal fish and their partner were out together (collective movements), when focal fish were still out of cover after a collective trip but their partner was already back under cover (returns) and focal fish going out and back under cover during solo trips. Average scores are shown with SEs. The distance of focal fish to their partner's lane during collective movements is depicted in white.

Focal fish swam on average in the middle of their compartment when in isolation (1.35 ± 3.35 mm; *t*_37_ = −0.41, *P* = 0.688; Figs. 2 and 3), but were on average much closer to their partner's compartment (39.1 ± 3.1 mm away; 35.9 mm from the middle) when paired (*t*_33_ = 6.47, *P* < 0.001). During the pairing stage, shy focal fish swam much closer to the adjacent compartment than bold focal fish (*t*_23.74_ = 4.05, *P* = 0.003; Fig. 1b). These results were not simply a consequence of the different partitions used in the isolation and pairing stages. Fish from the transparency control group, which were tested with a transparent partition and an empty adjacent compartment during the isolation stage, swam on average in the middle of their lane during the isolation stage (0.4 ± 7.6 mm; *t*_5_ = 0.04, *P* = 0.969). Their distance from the partition was not significantly different from the distance kept by focal fish during the isolation stage of the main experiment with an opaque partition (*t*_7.1_ = −0.20, *P* = 0.847). Furthermore, during the pairing stage, fish in the control group swam closer to the partition than during the isolation stage (*t*_4_ = 5.43, *P* = 0.006), even though they had a transparent partition throughout.

### Social Attraction: Collective States

During the pairing stage, fish in adjacent compartments (a ‘pair’) could be in different collective states: focal fish and their partner could both be out of cover (collective movement) or focal fish could be out alone during the initiation of these collective movements (initiation), during the return of these trips terminated by the partner (return) and during solitary trips (solo trip). During collective movements, focal fish stayed close to their partner's compartment, on average swimming 32.2 ± 2.7 mm from the transparent partition, and were consistent in their tendency to do so across both days (*r*_s_ = 0.48, *N* = 22, *P* = 0.024), providing the social attraction score for each fish. Social attraction was negatively related to the focal fish's own boldness (*F*_1,37_ = 20.82, *P* < 0.001) as well as that of their partner (*F*_1,37_ = 5.67, *P* = 0.023; full model: *F*_2,37_ = 11.55, *P* < 0.001; *R*^2^ = 0.39; Fig. 2). In other words, fish were less inclined to swim near their partner's compartment when they were bolder themselves or when paired with a bolder partner. Furthermore, the boldness of the partner had a positive effect on the total number of collective movements (i.e. trips when both fish were out together; *r*_s_ = 0.45, *N* = 48, *P* = 0.001). The average distance of focal fish to their partner's compartment did not differ significantly between trips initiated by the focal fish (i.e. when leading; 37.7 ± 4.4 mm) and those initiated by their partner (i.e. when following; 33.9 ± 3.2 mm; *t*_34_ = 0.69, *P* = 0.494).

When focal fish were out of cover alone, their average position was still significantly different from the middle of their lane and closer to the partner's compartment (49.9 ± 4.7 mm; *t*_39_ = −5.34, *P* < 0.001). This tendency to approach the partner's compartment when focal fish were out of cover alone was negatively related to their own boldness (*F*_1,38_ = 11.67, *P* = 0.002, *R*^2^ = 0.24), but, in contrast to the results obtained for collective movements, not related to the boldness of their partner (*F*_1,38_ = 1.60, *P* = 0.214).

Looking at the average lateral position of focal fish (the position relative to the partition separating the compartments), there was no significant difference in position during collective movement and the return from such movements (*V* = 340, *P* = 0.478; Fig. 3). However, focal fish tended to be further away from their partner's compartment during the initiation of collective movements (*V* = 407, *P* = 0.062; Fig. 3) and during solo trips (*V* = 435, *P* < 0.001; Fig. 3). Focal fish swam significantly closer to their partner's compartment when initiating collective movements than when on solo trips (*V* = 315, *P* = 0.035; Fig. 3). Even when we restricted our analysis to the first 10 cm out of cover, which was the average distance focal fish moved before being joined by their partner, we still found a trend for this effect (*V* = 303, *P* = 0.066). Focal fish swam significantly less far out of cover when they went out of cover alone on a solo trip (22.3 ± 2.0 cm) than when their partner was also out (i.e. during collective movements; 28.4 ± 1.3 cm; *t*_32_ = 4.95, *P* < 0.001).

### Collective Behaviour

While during the isolation stage fish in adjacent compartments were rarely out together (4.2 ± 1.0% of time out), during the pairing stage, when fish in adjacent compartments could see and interact with one another through the transparent partition, they spent considerably more time out together (26.9 ± 3.4% of time out; *V* = 75, *P* < 0.001). Furthermore, during the pairing stage fish had higher behavioural coordination with one another (0.74 ± 0.04; *V* = 230, *P* = 0.006) than when they were tested in isolation (0.59 ± 0.04). Pairs that had higher overall social attraction (i.e. pairs in which both fish swam closer to one another during collective movements) showed more coordinated behaviour (*r*_s_ = −0.38, *N* = 39, *P* = 0.018), but tended to travel shorter distances out of cover (*r*_s_ = 0.31, *N* = 39, *P* = 0.052). More sociable individuals led fewer trips (*F*_1,37_ = 7.96, *P* = 0.008, *R*^*2*^ = 0.18), with no effect of the sociability of the partner (*F*_1,36_ = 0.79, *P* = 0.381).

## Discussion

By tracking the movements of interacting pairs of three-spined sticklebacks, we found that individuals varied consistently in the average distance they maintained from their partner's compartment during collective movements, an aspect of their behaviour that we refer to as ‘social attraction’. Although less social individuals led more collective trips, pairs consisting of more social individuals showed higher behavioural coordination. The tendency for social attraction was negatively correlated with both the boldness of the focal fish and that of its partner, i.e. bolder fish with bolder partners stayed further away from their partner's lane, whereas shyer fish with shyer partners swam closer to it. Individuals that displayed stronger social attraction even persisted in swimming closer to their partner's lane when they were out of cover alone while their partner remained under cover. This result was not a consequence of using a transparent partition, since fish in the control group only swam closer to the transparent partition when the adjacent compartment contained another fish.

By investigating sociability behaviour in a dynamic social context, we have shown that social feedback plays a key role in social attraction tendencies and thereby affects group cohesion and coordination. Individuals with higher social attraction swam closer to one another, decreasing the distance between them (see also Cote et al., 2012), and because of the resulting positive feedback, became even more cohesive as a pair. At the same time, fish paired with bolder, less social partners increased their time spent out of cover (see also Harcourt et al., 2009; Jolles et al., 2014; Pike et al., 2008; Nakayama et al., 2012), but showed lower social cohesion because of less social feedback when approaching their partner. Not only do these findings substantiate the importance of social feedback in collective behaviour, as previously shown for boldness and leadership (Harcourt et al., 2009; Jolles et al., 2014; Nakayama et al., 2013; Pettit et al., 2013; Ward et al., 2013), they also support theoretical expectations about the consequences of personality differences for the functioning and structure of social groups (Wolf & Krause, 2014).

Our findings about the role of sociability expression in group coordination and cohesion are interesting in relation to leadership behaviour. Although pairs with higher overall social attraction had higher behavioural coordination, they tended to explore less far out of cover. Furthermore, individual social attraction was negatively correlated with the leadership tendencies of individuals. Previous studies have shown that bolder fish more often take the lead and that shyer fish more often follow (Harcourt et al., 2009; Jolles et al., 2014; Nakayama et al., 2013). Furthermore, bolder individuals often have fewer and weaker social interactions with conspecifics than do shyer conspecifics (Croft et al., 2009; Pike et al., 2008). Since boldness and social attraction were negatively linked in our study, our results suggest that bolder, less sociable individuals may often lead simply because they are less reluctant to move away from their partners, whereas shyer, more sociable, individuals become followers because they prioritize staying close to others (see also Couzin et al., 2005).

Our study is the first to show a direct negative impact of boldness on social attraction by assessing both in the same, ecologically relevant setting. This negative relationship is in line with most previous work that examined sociability and boldness in separate contexts (e.g. Budaev, 1997; Croft et al., 2009; Pruitt et al., 2010; Ward, Thomas, Hart, & Krause, 2004; but see Cote, Dreiss, & Clobert, 2008; Cote, Fogarty, Weinersmith, et al., 2010; Irving & Brown, 2013), and supports the idea of a more general difference in sociality between bold and shy individuals (see Magnhagen, 2012). For example, bold individuals have been shown to group less (Bell & Sih, 2007), to interact less regularly with their group mates (Croft et al., 2009; Pike et al., 2008), and to be either less reliant on social information (Harcourt et al., 2010; Kurvers, van Oers, et al., 2010) or to use it more to avoid conspecifics (Trompf & Brown, 2014). Together, these findings suggest that sociability and boldness may represent different aspects of the same, fundamental behavioural syndrome (see also Cote & Clobert, 2007; Sih, Cote, Evans, Fogarty, & Pruitt, 2012), with potentially large ecological implications (Sih et al., 2012; Webster & Ward, 2011). Differences in boldness and sociability may be expressions of underlying risk-prone or risk-averse behavioural types, as risk-averse individuals may be more motivated to group and to respond to conspecifics in order to lower their risk of predation (Krause & Ruxton, 2002). Together these findings show that differences in leadership behaviour may ultimately emerge from a combination of initial differences in the use of coordination rules and the resulting social feedback. This raises the intriguing idea that differences in personality between individuals may be maintained in populations because of their role in promoting social coordination (Johnstone & Manica, 2011; King et al., 2009).

To conclude, by tracking the movements of interacting pairs of fish in an ecologically relevant setting in which fish had to make decisions regarding exploration versus resting under cover, we have shown that social attraction, like leadership, is associated with individual boldness, but is also subject to social feedback, being influenced by the personality of the partner as well as that of the focal individual. Further studies are needed to better understand how boldness and social attraction vary in larger, more dynamic groups and how the composition of behavioural types ultimately affects group functioning and success.
